# Dissociable impacts of physical and psychological factors on side effects after COVID-19 vaccination in Japan: A within-subject repeated measures design

**DOI:** 10.1186/s40359-025-03325-4

**Published:** 2025-09-01

**Authors:** Kosuke Tsurumi, Hironobu Fujiwara, Teruhisa Uwatoko, Toshiya Murai

**Affiliations:** 1 Department of Psychiatry, Graduate School of Medicine, Kyoto University, 54, Shogoin-Kawahara-cho, Sakyo-ku, Kyoto, 606-8507 Japan; 2 Decentralized Big Data Team, RIKEN Center for Advanced Intelligence Project, 1-4-1 Nihombashi, Chuo-Ku, Tokyo, 103-0027 Japan; 3 The General Research Division, Center On Ethical, Legal and Social Issues, Osaka University Research, 2-8 Yamadaoka, Suita, Osaka 565-0871 Japan; 4 University Health Center, Kyoto University of Education, 1, Fukakusa-Fujinomori-Cho, Fushimi-Ku, Kyoto, Kyoto Japan

**Keywords:** COVID-19, Vaccine, Side effect, Nocebo effect, Depression, Anxiety

## Abstract

**Background:**

Although vaccination has significantly contributed to controlling the coronavirus disease 2019 (COVID-19) infections, a high risk of side effects has resulted in vaccination hesitancy. Nevertheless, as COVID-19 has become common but still has a severe impact on vulnerable people, we might need to follow a long-term vaccination routine. Therefore, elucidating the characteristics of individuals who may exhibit either vulnerability or resilience to the side effects associated with COVID-19 vaccinations is of the utmost importance for the vaccination plan against COVID-19.

**Methods:**

Self-reported data on demographics, physical health, addictive lifestyle habits, mental health, and side effects were collected online. For 998 participants who received two vaccination doses, we performed repeated measures ANOVA, including side effects scores as dependent variables and individual characteristics as independent variables.

**Results:**

We found interactions between vaccine dose and history of suspected lifestyle diseases (F = 4.460, *p* = 0.012) or trait anxiety (F = 5.548, *p* = 0.019). Besides the aggregation of side effects after the second dose compared to the first dose, we found that increased side effects after vaccination were associated with specific personal factors: younger age (F = 18.973, *p* = 0.000), female sex (F = 34.507, *p* = 0.000), low BMI (F = 4.205, *p* = 0.015), no history of “specific health guidance” (F = 5.004, *p* = 0.007) or smoking (F = 9.123, *p* = 0.003) or drinking (F = 3.335, *p* = 0.036), and higher levels of depressive symptoms (F = 15.134, *p* = 0.000) or trait anxiety (F = 26.350, *p* = 0.000).

**Conclusion:**

Physical characteristics and lifestyle habits linked with intact immune function enhanced side effects, whereas psychological characteristics linked with impaired immune function also enhanced side effects. These seemingly contradictory results could be explained at least partly by nocebo effects and suggest the need for psychological support for people with high levels of depressive symptoms or trait anxiety. Furthermore, interaction results imply that such support might be especially beneficial to those who are young and unaffected by lifestyle diseases while simultaneously showing high trait anxiety.

**Supplementary Information:**

The online version contains supplementary material available at 10.1186/s40359-025-03325-4.

## Introduction

The coronavirus disease 2019 (COVID-19) rapidly spread worldwide and had a profound impact on our daily lives. Although vaccination has contributed significantly to the control of infections [1], newly developed vaccines can induce a range of nonspecific side effects, such as fever, headache, and fatigue [2]. Because the underlying mechanisms responsible for these side effects remain unclear, a substantial number of individuals exhibit hesitancy towards being vaccinated. However, the persistent number of new hospitalizations, even to intensive care units, by COVID-19 [3] indicates that COVID-19 remains a threat to vulnerable people. Accordingly, we might need to adopt a similar approach to COVID-19 vaccination as we do in terms of influenza, which involves continuing efforts to maintain vaccinations in response to the fluctuating and ever-changing situation over time,　especially for high-risk populations such as children, elderly, those with chronic medical conditions and health workers [4]. Nevertheless, COVID-19 vaccination exhibits an increased risk for side effects compared to other vaccinations [5]. Therefore, elucidating the characteristics of individuals who exhibit vulnerability or resilience towards the side effects associated with COVID-19 vaccinations is of the utmost importance for the vaccination plan against COVID-19. 

Vaccines elicit the production of neutralizing antibodies, enhancing the resilience of vaccinated individuals against pathogens [6]. Conventional vaccines, such as those for influenza, use inactivated viruses or parts of viruses as antigens to produce neutralizing antibodies [6], whereas COVID-19 vaccines are designed to utilize mRNA to synthesize spike protein of the severe acute respiratory syndrome coronavirus 2 (SARS-CoV-2) and induce the production of neutralizing antibodies that target the spike protein [7]. Antibodies, as the final product of immune response [8], can be influenced in various ways by individual characteristics that impact the immune system. Thus, those characteristics could potentially affect both the anti-infection and the side effects of vaccination.

Various general individual physical characteristics have been recognized as affecting immune function. Notably, factors such as ageing [9], male sex [10], and obesity [11] have been associated with diminished innate and adaptive immune responses, rendering individuals more susceptible to infection. For example, ageing [12], male sex [13], and obesity [14] are associated with blunted immune responses following influenza vaccination. Thus, these factors may also impair the immune response after COVID-19 vaccination.

In addition, certain addictive lifestyle habits also affect immune systems. Chronic alcohol intake [15] and smoking [16] compromise both the innate and adaptive immune responses, making individuals more vulnerable to infectious diseases. For example, alcohol consumption is supposed to weaken the immune response after Hepatitis B vaccination [17], and smoking is shown to hinder the immune response after influenza [18] and hepatitis B [19] vaccination. Accordingly, drinking and smoking habits could also decrease the immune response after COVID-19 vaccination.

Furthermore, individual psychological characteristics can impede immune function. Depression [20, 21] and anxiety [22, 23] alter innate and adaptive immune responses, thus increasing susceptibility to the vulnerability to infection. For example, caregiving of a family member with a disability, which is a risk factor for depression and anxiety, hinders the immune response after influenza [24, 25] and pneumococcal [26] vaccination. Therefore, depressive symptoms and anxiety may decrease the immune response after COVID-19 vaccination. Nevertheless, only a limited number of studies have simultaneously investigated the effects of psychological and physical factors on the side effects of COVID-19 vaccination [27]. Furthermore, no study has comprehensively explored the impact of personal factors, including demographic, psychological, physical variables, and addictive lifestyle habits, on the side effects.

On the other hand, a history of influenza infection is known to increase the immune response after influenza vaccination [28]. Healthcare workers at increased risk of infection may have an enhanced immune response after COVID-19 vaccination. Furthermore, although repeated vaccinations might be necessary for infection control, the side effects after COVID-19 vaccination are known to become more severe after the second dose than the first dose [2, 29]. However, no study has as yet investigated the individual characteristics that contribute to the exacerbation of dose-dependent side effects after COVID-19 vaccination.

Accordingly, we examined the influence of these multifaceted general personal factors and vaccine doses on self-reported side effects following COVID-19 vaccination. We hypothesized that vaccine dose, infection history, being a healthcare worker, and specific individual characteristics associated with an intact immune response, such as young age, female sex, optimal nutritional status, absence of drinking or smoking habits, and low levels of depressive symptoms or anxiety, could exaggerate side effects following COVID-19 vaccination.

## Methods

### Participants and procedure

1458 Japanese volunteers participated in this study via the web-based platform *Google Form* (Google LLC, CA). Participants were recruited by email, flyers, word-of-mouth, and online advertisements via social media from July 2021 to January 2022 (from just before the fifth wave to the beginning of the sixth wave of the COVID-19 pandemic in Japan). Upon accessing the provided URL, participants found an explanation of the study on the first page, and then they proceeded to the survey after providing their consent. The survey contained self-reported inquiries related to demographic data, addictive lifestyle habits, psychological variables, COVID-19 infection history, and the vaccine dose. Unvaccinated participants completed the investigation by stating their vaccine dose as zero and their reasons for remaining unvaccinated. Vaccinated participants reported the extent of their side effects experienced after each dose. All responses remained anonymous, were securely stored, and were accessible solely to the authors. We included participants who received two doses of the vaccine, were aged 18 years or older, and whose side effect scores could be calculated. We excluded participants who received only one dose of the vaccine, did not receive the vaccine, were under 18 years of age, and whose total side effect scores could not be calculated. We also excluded those assumed to have engaged in survey satisficing. We considered participants to have engaged in survey satisficing if they had a CAGE score of one or more (i.e., they had a drinking habit), although they reported no drinking history, or if they reported all the side effect scales in the same score. This study was approved by the ethics committee of the Kyoto University Graduate School and Faculty of Medicine and was conducted in accordance with the guidelines of the Declaration of Helsinki.

### Instruments

Demographic data included age, sex, occupation, height, body weight, and history of “specific health guidance”. Age was stratified into categories based on previous studies. In view of the markedly higher mortality rate of influenza reported in individuals aged 65 years or older [30] and the pronounced immunosenescence observed in this age group [31, 32], this group was classified as a specific subgroup. Additionally, considering the increased prevalence of lifestyle diseases, such as diabetes, from the age of 40 years [33], and the reduced immune responses identified in this age group [34], individuals younger than 40 years were classified as a separate category. Participants were therefore stratified into three subgroups: adolescents and young adults (< 40), middle-aged adults (40–64), and older adults (≧65). Information about occupation was acquired in a binary-choice format (healthcare worker or non-healthcare worker), after which participants reported their specific occupations. Body Mass Index (BMI) was calculated from height and weight and categorized into these three subgroups: underweight (< 18.5), standard weight (18.5—24.9), and obesity (≧25) based on the guidelines of the Japan Society for the Study of Obesity [35, 36]. The BMI cut-off point is different from that of Western countries based on the evidence that despite a lower obesity rate [37], East Asians showed a higher risk for lifestyle diseases, such as diabetes mellitus [38, 39], hypertension [39], and fatty liver [40] compared to Caucasians. “Specific health guidance” is designed to screen metabolic syndrome as an additional part of Japanese health checkups. Individuals aged 40 years or older who are public national health insurance subscribers undergo assessments of abdominal circumference, blood lipid and sugar levels, and blood pressure during health checkups. Individuals exceeding the specified abdominal circumference (males: 85 cm, females: 90 cm), those having a BMI of 25 or higher, or those meeting at least one of these three criteria (abnormal blood lipid levels, blood sugar levels, or blood pressure) become subject to “specific health guidance”. The subjects for “specific health guidance” are regarded as high-risk populations for lifestyle diseases and are encouraged to experience intervention by a public health nurse or a dietitian. Regarding this “specific health guidance”, participants 40 years or older were categorized into these three subgroups: ‘had never been included in the guidance’, ‘had never been included in the guidance but had been informed of some issues during the health check system’, and ‘had been included in the guidance program’.

Information on addictive lifestyle habits consisted of smoking history (yes or no), drinking history (yes, no, or past drinker), and a CAGE questionnaire [41]. The CAGE questionnaire consists of four items designed to screen for alcohol problems. Participants respond with either a ‘yes’ or ‘no’ to each item, and if they answer positively to two or more items, they may have the possibility of alcohol dependence.

Psychological assessment was conducted using the 2-item Patient Health Questionnaire (PHQ-2) [42] and the trait component of the State-Trait Anxiety Inventory–Form JYZ (STAI) [43]. PHQ-2 is a short form of PHQ-9 [44] designed to evaluate depressive symptoms. It consists of two items rated on a 4-point Likert scale to screen depression mainly in primary-care settings. The total score range for PHQ-2 is 0–6, with a cut-off score of ≧ 3, indicating a potential for depression. Despite its brevity, PHQ-2 exhibits sufficient sensitivity (0.77) and specificity (0.95) for screening depression [42]. The trait component of STAI (STAI trait) consists of 20 items rated on a 4-point Likert scale and assesses individual variations in trait anxiety. The total score range for the STAI trait is 20–80. The STAI trait demonstrates good internal consistency (Cronbach’s alpha of 0.896 for males and 0.904 for females) and satisfactory retest reliability (Pearson’s correlation coefficients of 0.856 for males and 0.642 for females) [43]. Considering sex differences in trait anxiety [45], subjects were stratified into higher or lower levels of trait anxiety groups using the median-splitting method for each sex. We categorized the above individual characteristics into two or three groups to examine the interaction between these characteristics and dose on side effects.

Side effect symptoms following COVID-19 vaccination were selected with reference to a previous study [2]. In that study, side effects were categorized into systemic and local manifestations. Systemic side effects included headache, fatigue, chills and shivering, diarrhea, fever, arthralgia, myalgia, and nausea. Local side effects included local pain, swelling, tenderness, redness, itch, warmth, bruising, and swollen armpit glands. Each side effect was assigned a score based on its severity and duration: 0 indicated the absence of symptoms; 1 denoted mild symptoms that disappeared within 48 h, 2 represented moderate or severe symptoms that subsided within 48 h, and 3 indicated moderate or severe symptoms that were resolved within one week. The total score range for each vaccine dose was 0–48.

### Statistical analysis

The data were analyzed with SPSS version 25 (IBM, USA). Repeated measures ANOVA was conducted with the total side effect score (i.e., the sum of systemic and local side effects, measured after both the first and second vaccine doses) as the dependent variable. The independent variables consisted of (a) vaccine dose (first vs. second; within-subject factor) and (b) age group, sex, BMI group, history of “specific health guidance,” smoking history, drinking history, possible alcohol dependence, depressive symptom level, and trait anxiety level (between-subject factors). For categorical between-subject factors with three subgroups (age, BMI, “specific health guidance” history, and drinking history), Tukey’s post hoc tests were performed with correction for multiple comparisons. This correction controls for inflated Type I error; therefore, only the differences that remained significant after adjustment were interpreted.

## Results

### Sample characteristics

**Fig. 1:**
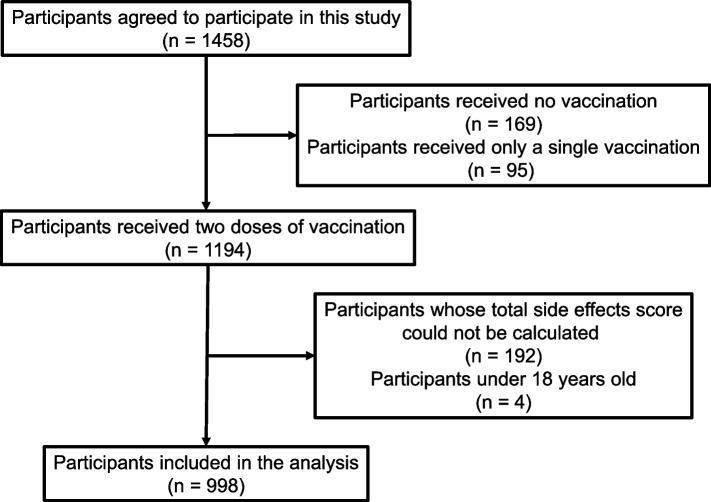
Flow diagram of the participants' selection process

Among the 1458 participants, 169 did not receive vaccination, and 95 received only a single dose, and therefore 1194 participants received both doses. However, 192 participants provided responses in unspecified phrases rather than with numerical values to at least one inquiry about side effects, leading to the inability to calculate their total side effect score. Furthermore, four participants were excluded because they were under 18 years old. Accordingly, data from 998 participants were analysed (Fig. 1). Among them, all without a drinking history scored 0 on CAGE, and no one scored all the side-effect scales in the same score. Accordingly, we regarded those participants kept sufficient attention throughout the survey. Two participants, who committed data entry mistakes as keeping their weight, were excluded from the ANOVA analysis involving BMI as the independent variable. Also, three participants who identified their sex as ‘other’ were excluded from the ANOVA analysis including sex or STAI-trait, where the cut-off score differed based on sex, as an independent variable due to the limited sample size of the ‘other’ category. Additionally, 41 participants who failed to declare whether they were or were not healthcare workers were excluded from the ANOVA analysis incorporating occupation as the independent variable. Regarding the ANOVA analysis incorporating the history of “specific health guidance” as an independent variable, participants under age 40 were excluded from the analysis because the guidance specifically targeted individuals aged 40 years or older. Due to the small number of participants with a history of COVID-19 infection (14 before vaccination, two between vaccinations, and two after vaccinations), we did not conduct an ANOVA analysis including infection history as an independent variable. Table 1 shows the total side effects score after the first and second vaccine doses for each group categorized by their respective characteristics. The prevalence of each side effect is summarized in Table S1. 

**Table 1:** Breakdown of the side effect scores divided by individual characteristics categories

		1 st dose			2nd dose		
		mean	SD	n	mean	SD	n
Age	< 40	6.51	6.26	398	9.65	7.68	398
	40 ~ 64	5.18	6.05	554	7.84	7.41	554
	≧65	2.40	3.64	45	3.33	4.49	45
Sex	male	4.20	4.59	337	6.59	6.49	337
	female	6.23	6.50	658	9.21	7.77	658
Occupation	healthcare	4.81	5.45	244	7.14	6.55	244
	non-healthcare	5.83	6.35	713	8.86	7.91	713
BMI	< 18.5	7.01	6.29	87	10.48	8.13	87
	18.5 ~ 24.9	5.46	5.62	683	8.38	7.24	683
	≧25	5.46	7.33	226	7.57	8.09	226
History of “specific health guidance”	no guidance	5.32	6.45	367	8.27	7.88	367
	pointed out some issues	4.65	5.06	96	7.49	6.83	96
	included into guidance	4.31	5.01	137	5.54	5.66	137
History of smoking	never	5.90	6.42	738	8.75	7.64	738
	past or now	4.70	5.06	260	7.29	7.17	260
History of drinking	never	6.37	7.20	273	9.01	7.96	273
	past	5.63	5.95	203	8.70	7.36	203
	now	5.17	5.50	522	7.91	7.37	522
CAGE	normal	5.69	6.20	907	8.50	7.67	907
	alcohol problem	4.59	5.09	91	7.05	6.03	91
STAI-trait	low	4.78	5.30	480	7.09	7.04	480
	high	6.26	6.50	515	9.47	7.67	515
PHQ-2	low	5.32	5.87	877	8.08	7.38	877
	high	7.53	7.42	121	10.48	8.38	121

*Abbreviations: BMI* body mass index, *STAI* state-trait anxiety inventory, *PHQ* Patient Health Questionnaire

^*^p < 0.05

### ANOVA results

#### Interaction between dose and individual characteristics

Significant interactions were found between the total side effect scores of each dose and age, history of “specific health guidance”, or anxiety scores (Table 2). In short, younger age, no history of “specific health guidance”, and higher trait anxiety exaggerated the elevation of side effects after the second vaccination dose compared to the first dose.

**Table 2:** Results of repeated measures ANOVA analysis for side effect scores

			ANOVA-interaction				
	number of categories		MS	df	*F*	*p*	Partial *η 2*
Age	3	age x dose	53.632	2	2.926	0.054	0.006
		age	1365.852	2	18.652	0.000*	0.036
		dose	853.753	1	46.584	0.000*	0.045
Sex	2	sex x dose	39.371	1	2.142	0.144	0.002
		sex	2409.362	1	33.987	0.000*	0.033
		dose	3201.335	1	174.205	0.000*	0.149
Occupation	2	occupation x dose	44.041	1	2.389	0.123	0.002
		occupation	682.066	1	8.896	0.003*	0.009
		dose	2611.379	1	141.650	0.000*	0.129
BMI	3	BMI x dose	39.498	2	2.147	0.117	0.004
		BMI	322.822	2	4.279	0.014*	0.009
		dose	2072.353	1	112.631	0.000*	0.102
History of “specific health guidance”	3	“specific health guidance” x dose	77.026	2	4.521	0.011*	0.015
		“specific health guidance”	351.158	2	4.948	0.007*	0.016
		dose	1206.719	1	70.820	0.000*	0.106
History of smoking	2	history of smoking x dose	6.555	1	0.356	0.551	0.000
		history of smoking	679.333	1	9.029	0.003*	0.009
		dose	2842.866	1	154.215	0.000*	0.134
History of drinking	3	history of drinking x dose	5.847	2	0.317	0.728	0.001
		history of drinking	247.115	2	3.273	0.038*	0.007
		dose	3405.032	1	184.577	0.000*	0.156
CAGE	2	CAGE x dose	5.127	1	0.278	0.598	0.000
		CAGE	267.979	1	3.542	0.060	0.004
		dose	1150.715	1	62.417	0.000*	0.059
STAI-trait	2	STAI-trait x dose	101.362	1	5.535	0.019*	0.006
		STAI-trait	1848.278	1	25.866	0.000*	0.025
		dose	3785.045	1	206.671	0.000*	0.172
PHQ-2	2	PHQ-2 × dose	1.963	1	0.106	0.744	0.000
		PHQ-2	1127.227	1	15.072	0.000*	0.015
		dose	1732.590	1	93.963	0.000*	0.086

*Abbreviations BMI* body mass index, *STAI* state-trait anxiety inventory, *PHQ* Patient Health Questionnaire

^*^p < 0.05

#### Main effect of dose

A significant main effect of dose was observed across all independent variables, including age, sex, occupation, BMI, history of “specific health guidance”, smoking history, drinking history, CAGE, PHQ-2, and STAI-trait (Table 2). Briefly, the second dose led to significantly more pronounced side effects than the first dose in all ANOVAs.

#### Main effect of individual characteristics

A significant main effect was observed for age, sex, occupation, BMI, history of “specific health guidance”, history of smoking, history of drinking, PHQ-2, and STAI-trait. Additionally, a marginally significant main effect was found for the risk of drinking (Table 2).

Regarding individual characteristics with two categories, it was revealed that females (vs males), non-healthcare workers (vs healthcare workers), individuals with no history of smoking (vs those with a history of smoking), individuals with higher levels of depressive symptoms (vs those with lower levels of depressive symptoms), and those with higher levels of trait anxiety (vs those with lower levels of trait anxiety) experienced more pronounced side effects. Moreover, individuals with a low-risk drinking profile exhibited slightly harsher side effects than those with a high-risk profile.

For individual characteristics with three categories, post hoc analysis was conducted to identify the group that exhibited significantly more side effects compared to the others. Post hoc analysis of the age group revealed that younger subjects experienced significantly more severe side effects than middle-aged subjects and elderly subjects, while middle-aged subjects showed significantly harsher severe side effects than elderly subjects. Based on the post hoc analysis of BMI, individuals with a low BMI experienced significantly more severe side effects than those with a normal BMI and a high BMI. In the case of the post hoc analysis of the history of “specific health guidance”, subjects who had never received it encountered significantly more severe side effects than those who had previously received it. The post hoc analysis of drinking habits indicated that individuals who had never consumed alcohol displayed significantly more substantial side effects than those with drinking habits (Table 3).

**Table 3:** Results of Tukey’s post hoc analysis for multiple comparisons

						95% Confidence Interval	
	A	B	Mean Difference (A – B)	Standard Error	*p*	Lower Bound	Upper Bound
Age	< 40	40 ~ 64	1.57	0.398	0.000*	0.64	2.50
		≧65	5.21	0.952	0.000*	2.98	7.45
	40 ~ 64	≧65	3.65	0.938	0.000*	1.44	5.85
BMI	< 18.5	18.5 ~ 24.9	1.83	0.699	0.025*	0.19	3.47
		≧25	2.23	0.775	0.011*	0.41	4.05
	18.5 ~ 24.9	≧25	0.40	0.471	0.667	−0.70	1.51
History of “specific health guidance”	no guidance	some issues pointed out	0.73	0.683	0.538	−0.88	2.33
		included into guidance	1.87	0.596	0.005*	0.46	3.27
	pointed out some issues	included into guidance	1.14	0.793	0.322	−0.72	3.00
History of drinking	never	past	0.53	0.569	0.621	−0.81	1.87
		now	1.15	0.459	0.032*	0.08	2.23
	past	now	0.62	0.508	0.437	−0.57	1.82

*Abbreviation: BMI* body mass index

^*^p < 0.05

Some may argue that the greater severity of side effects among non-healthcare workers could be attributed to other individual variables. Notably, non-healthcare workers consisted of a larger proportion of females (chi-square test, *p* = 0.000). However, even after controlling for sex, the difference between non-healthcare workers and healthcare workers remained statistically significant (ANCOVA, *p* = 0.006).

## Discussion

In our study, no history of “specific health guidance” and higher trait anxiety exaggerated the elevation of side effects after the second vaccination dose compared to after the first. Overall, self-reported side effects were more pronounced after the second dose of COVID-19 vaccine compared to after the initial dose. Additionally, younger subjects (vs middle-aged to older subjects), middle-aged subjects (vs older subjects), females (vs males), non-healthcare workers (vs healthcare workers), individuals with lower BMI (vs normal to higher BMI), those who had never received the “specific health guidance” (vs those who had received it), those without a history of smoking (vs those with a history of smoking), those who had never consumed alcohol (vs those with drinking habits), those with higher levels of depressive symptoms (vs those with lower levels of depressive symptoms) and those with higher levels of trait anxiety (vs those with lower levels of trait anxiety) exhibited severer self-reported side effects.

In summary, besides the aggravation of side effects after the second dose, as hypothesized, individuals with immune-enhancing physical factors, such as younger age, female sex, low BMI, or no history of “specific health guidance”, reported more severe side effects. Among these factors, history of “specific health guidance” showed an interaction with the vaccine dose, wherein no history of “specific health guidance” exaggerated the elevation of side effects after the second dose of the COVID-19 vaccine compared to the first dose. Similarly, individuals with immune-enhancing lifestyle habits (i.e. no history of smoking or drinking) experienced heightened side effects. Conversely, contrary to our hypothesis, individuals with immune-suppressing psychological factors (higher levels of depressive symptoms or trait anxiety) showed more pronounced side effects. Among these factors, trait anxiety displayed an interaction with the vaccine dose, resulting in higher levels of trait anxiety exacerbating the elevation of side effects after the second COVID-19 vaccination compared to the first.

### Relationship between dose and side effect score

Self-reported side effects were more pronounced following the second dose of COVID-19 vaccine compared to after the initial dose. This observation is consistent with previous studies [29, 46]. Furthermore, a history of COVID-19 infection has been shown to amplify the side effects of COVID-19 vaccination [2, 29, 47, 48]. These phenomena could be explained by the general principle that repeated exposure to the same antigen enhances the immune response [49].

### Relationship between physical factors and side effect score

Females reported more pronounced side effects after COVID-19 vaccination compared to males, a finding in line with previous studies [2, 46, 50–54]. Besides, females are known to have higher antibody levels after COVID-19 vaccination [54–56]. Thus, the female sex might exaggerate both the anti-infection effect and the side effects after COVID-19 vaccination. On the other hand, males show abated immune responses [10], making them more vulnerable to infection [13], and they generally show diminished immune response and attenuated side effects after vaccinations other than for COVID-19 [10]. These phenomena could be supported by the fact that numerous immune-related genes are located on the X chromosome [57]. Overall, an intact immune system based on the female sex might contribute to robust immune responses and more severe adverse effects after COVID-19 vaccination, and vice versa for the male sex.

Persons with relatively immune-enhancing physical factors such as younger age, low BMI and no history of “specific health guidance” reported more pronounced side effects after COVID-19 vaccination. These observations are consistent with previous studies, which showed enhanced side effects after COVID-19 vaccination among individuals of a younger age [27, 46, 50–53, 58–61] or with a standard weight [27, 60, 62] compared to those of older age or those overweight, respectively. Besides, individuals of a younger age [2, 46, 50–53, 59, 61], lower BMI [55], or smaller waist circumference [63] exhibited higher antibody levels after COVID-19 vaccination than those of older age, higher BMI, or larger waist circumference. These findings suggest that age and obesity could influence anti-infection efficacy and side effects after COVID-19 vaccination in parallel.

In addition, elderly and obese individuals exhibit a diminished immune response [9, 11], making them susceptible to infection [11, 64], and they also demonstrated reduced antibody levels after vaccinations other than for COVID-19 [64, 65]. Altogether, an intact immune system based on health-beneficial factors could result in both robust immune responses and more severe adverse effects after COVID-19 vaccination. Conversely, these effects could be abated by health-detrimental factors.

### Relationship between addictive lifestyle habits and side effect score

Addictive lifestyle habits such as smoking and drinking were associated with reduced side effects after COVID-19 vaccination. This observation was consistent with previous studies regarding smoking [62]. In addition, drinking [66, 67] and smoking [63, 67, 68] have been found to lower antibody levels after COVID-19 vaccination. Taken together, drinking and smoking could exert a parallel impact on both anti-infection efficacy and side effects after COVID-19 vaccination.

Furthermore, individuals with chronic drinking [15] and smoking [16] show a diminished immune response, rendering them more susceptible to infections. Additionally, excessive drinking [69] and smoking [18, 19] hinder antibody production following vaccination against diseases other than COVID-19. Overall, a dysregulated immune system resulting from drinking and smoking could mitigate in parallel both appropriate immune responses and severe adverse effects after COVID-19 vaccination. The reduction in both immune responses and side effects following COVID-19 vaccination due to health-detrimental life habits, such as drinking and smoking, appear to be two sides of the same coin, juxtaposing with the enhancement of immune responses and side effects after COVID-19 vaccination resulting from health-promoting factors including young age, optimal nutritional status and a robust immune system in females.

### Relationship between psychological factors and side effect score

Greater depressive symptoms and trait anxiety were associated with more serious side effects after COVID-19 vaccination. These findings align with previous studies on clinical populations [27]. On the other hand, depressive symptoms have been associated with a blunted antibody level after COVID-19 vaccination [70]. Additionally, individuals with depression [21] and anxiety disorders [22] are known to show decreased immune function, making them more vulnerable to infections. Considering the association between reduced immune function and diminished side effects after COVID-19 vaccination observed in individuals with various physical characteristics or addictive lifestyle habits (old age, male sex, obesity, drinking habits, smoking history), the exaggerated side effects among individuals with immune-suppressing factors, such as heightened levels of depressive symptoms and trait anxiety, seem to be controversial.

We assume that these increased side effects among these populations could be at least partly ascribed to the nocebo effect, as is prominently observed following COVID-19 vaccination [71]. The nocebo effect is recognized as being associated with somatization [72], a condition characterized by the presence of excessive somatic symptoms beyond what would be expected based on organic disturbances [73]. Somatization is now listed in DSM-5 as “Somatic Symptom and Related Disorders” [74] and exhibits a close association with depression and anxiety [75–77]. Accordingly, elevated side effects among participants with higher depressive symptoms and trait anxiety could be attributed to somatization emerging as the nocebo effect. Taken together, the impact of high levels of depressive symptoms and anxiety on the intensification of side effects by the nocebo effect might have outweighed that on the reduction in side effects via impaired immune function. From another point of view, the nocebo effect observed in individuals with higher depressive symptoms and trait anxiety could be alleviated through interventions aimed at addressing depressive symptoms and anxiety. Accordingly, psychological support to alleviate depressive symptoms and anxiety, including psychotherapy and the provision of pertinent information, could potentially alleviate side effects after COVID-19 vaccination in individuals with higher levels of depressive symptoms and anxiety.

### Relationship between healthcare profession and side effect score

Non-healthcare workers reported more pronounced side effects, even after controlling for sex, which could influence anxiety levels [45]. This disparity could partly be attributed to the dynamic nature of state anxiety. Healthcare workers, who face an elevated risk of exposure to the novel coronavirus, might appreciate the anti-infection effect of vaccination, which may result in a reduction of state anxiety and subsequent alleviation of nocebo effects. Moreover, the comprehensive knowledge and understanding of vaccines by healthcare workers could contribute to decreased state anxiety and the mitigation of nocebo effects. However, it should be noted that we utilized the measure of trait anxiety instead of state anxiety in our test battery, thereby precluding further discussion on this issue. On the other hand, healthcare workers experienced intense stress due to stringent infection control measures and increased workloads during the pandemic [78]. Although this stress could exacerbate anxiety and depression, healthcare workers showed lower anxiety and depression scores compared to non-healthcare workers. One explanation for this phenomenon could be the delay in the approval and distribution of COVID-19 vaccines observed in Japan [79]. The delay in vaccination might have fostered increased positive expectancy regarding the vaccine’s protective effect, and the prioritization of healthcare workers for vaccination could have alleviated their anxiety about infection. This mitigating effect could have outweighed the impact of stress among healthcare workers, leading to a compromised nocebo effect. Because the absolute number of participants with a history of COVID-19 infection was small, the influence of infection history in healthcare workers was negligible, possibly leading to a clear manifestation of the nocebo effect in non-healthcare workers.

### Limitation and strength

There are several limitations to our study. Firstly, the side effects score adopted in this study holds some arbitrariness by nature. Thus, individual attitudes toward the severity of side effects might have influenced the side-effect score. Secondly, because all questionnaires relied on self-reports, the scores obtained could be inherently subjective. Moreover, the lack of objective measures of the immune system due to the online survey method precluded us from attributing the severity of side effects entirely to immunity enhancement or suppression associated with individual characteristics. Thirdly, the interpretation of our study was constrained by its cross-sectional design and the absence of a measure for state anxiety. Although a certain degree of association between state anxiety and trait anxiety [80] might support the possible influence of trait anxiety on nocebo effects, future studies need to employ a longitudinal design and incorporate measures of state anxiety to capture the dynamic nature of its impact on nocebo effects. Besides, a cross-sectional design may introduce recall bias, especially regarding side effects experienced after the first dose. This recall bias might have amplified our findings of the significant main effect of the vaccine dose. Fourthly, our study did not encompass all potential side effects, including those of a fatal nature. Therefore, our findings cannot be generalized to the influence of individual characteristics on fatal or other side effects. Fifthly, a small number of subjects with a history of COVID-19 infection precluded us from examining the effect of infection history on side effects. Sixthly, we did not include positive factors that might enhance immune function and reduce side effects. However, because positive and negative factors are the head and tail of the same coin, the influences of positive factors could be at least partly inferred from those of negative factors. For example, meditation, which can improve depressive symptoms and anxiety [81], may reduce side effects attributed to the nocebo effect. On the other hand, exercise, which can also improve depression and anxiety as well as obesity [81], could mitigate or enhance side effects by reducing the nocebo effect or improving immune function, respectively. Future studies should also consider these positive factors. Seventhly, the exploratory nature of this study and the correlation between independent variables precluded multiple comparisons and regression analysis. Our findings need to be replicated in future studies. Notwithstanding these limitations, our samples included individuals across a wide age range and from diverse occupations. Moreover, we incorporated comprehensive variables such as physical characteristics, addictive lifestyle habits, and psychological characteristics. Because these individual characteristics are commonly inquired about in various settings, such as health checkups, clinical situations, and surveys, individuals can roughly infer whether they are vulnerable or resilient to side effects after COVID-19 vaccination based on our results without additional effort.

## Conclusion

Our findings suggest that specific health-promoting physical factors and lifestyle habits, recognized for their ability to enhance immune functions, have the potential to amplify side effects after COVID-19 vaccination. However, it is crucial to note that the intensity of these side effects could reflect an intact immune response and therefore should not necessarily be regarded as a negative phenomenon. Conversely, individuals exhibiting higher levels of depressive symptoms and trait anxiety, despite their expected impaired immune function, reported elevated side effects. This exacerbation of side effects in individuals with higher depressive symptoms and trait anxiety could be attributed to nocebo effects. Therefore, these effects might be alleviated through psychological support, including psychotherapy and the dissemination of pertinent information concerning vaccination. Such supports might be especially beneficial to those who are young and unaffected by lifestyle diseases while simultaneously showing high trait anxiety. 

## Supplementary Information

Supplementary Material 1.

## Data Availability

The datasets used and/or analysed during the current study are available from the corresponding author on reasonable request.

## Abbreviations

COVID-19: The coronavirus disease 2019
SARS-CoV-2: The severe acute respiratory syndrome coronavirus 2
BMI: Body Mass Index
PHQ-2: The 2-item Patient Health Questionnaire
STAI: The State-Trait Anxiety Inventory–Form JYZ
